# Supplementary material for: Sleep prevents catastrophic forgetting in spiking neural networks by forming a joint synaptic weight representation
Source: PLoS Comput Biol. 2022 Nov 18;18(11):e1010628. doi: 10.1371/journal.pcbi.1010628 (PMC9674146; doi:10.1371/journal.pcbi.1010628)
Supplement: Attachment — Submitted filename: ReviewerResponse.docx [file pcbi.1010628.s007.docx]

Reviewer #1,

Thank you for your comments on our manuscript, we have taken them all into consideration and have made the appropriate revisions. With respect to your main concern:

“My main concern is a substantial proportion of the results are examples from a single experiment and sometimes a single neuron, with no suggestion of how well these findings generalise to other runs with other random seeds or initialisations. The manuscript would benefit from some indication of how robust these results are. For example, constructing a single metric to summarise the bias of the receptive fields in the output layer ne	urons towards the task-specific orientations (figure 2), and showing a distribution of these values across neurons and experiments rather than relying on a single example. This is particularly important if the authors do not intend to publicly share their code.”

We apologize that we did not make clear the robustness of our results. We have now revised the text in numerous places to emphasize that most of our analysis takes into account multiple trials differing in random seeds, particularly with respect to performance metrics (Fig 1 B,C; Fig 3 A-F; Fig 4 A,B; Sup 1 A,B; Sup 2 A-F; Sup 3 A,B; Sup 4 A-C). Additionally, we modified the manuscript to highlight that portions of the weight analyses were also averaged across trials with different random seeds (Fig 4 D; Fig 6 B, Fig 7 A - F). We have also changed subplots 4 - C and Sup 3 - C to include average histograms across trails to display how the qualitative results generalize across trials.

**Figure 4 C** -(Blue Bars - Single trial | Orange Line / Orange Shade - Mean / Std across 10 trials)

**Figure S3 C**-(Blue Bars - Single trial | Orange Line / Orange Shade - Mean / Std across 10 trials)

To address your specific concerns with respect to Figure 2 and 4, we have developed a metric that encapsulated the receptive field qualities we suggest are present and have plotted their distribution across trials for various training paradigms in Figure S5. The metric is described in a new section of the **Methods: Particle responsiveness metric**, and referenced throughout the text as stated below:

Line 714

***Particle responsiveness metric (PRM)***

*“ The particle responsiveness metric (PRM) developed to quantify how responsive the network’s weight matrix is to specific food particle orientations thereby allowing the quality of the receptive field for a given task to be determined was defined as follows:*

$$\mathrm{PRM}\left( \mathrm{ParticleType} \right)= \sum_{\forall O\in Output} \mathrm{grand}\left( \mathrm{DirectionMask}\left( O \right) ⨀\sum_{\forall H\in Hidden} W_{H\to O}*\sum_{\forall P\in ParticleMasks} \left( W_{H} ⨀ P \right)*grand\left( W_{H} ⨀ P \right)^{2} \right)$$

*Here* $Output$ *is the set of all output layer neurons,* $O$*;* $Hidden$ *is the set of all hidden layer neurons,* $H$*;* $ParticleMasks$ *is the set of masks,* $P$*, representing all possible locations of a single instance of a* $ParticleType$ *in the input field (e.g., horizontal bars would be a set of masks with a single horizontal bar placed in all possible locations in the visual field; each particle mask* $P$ *consists of a 7 x 7 matrix of zeros with ones being placed in locations that correspond to current food pixels).* $W_{H}$ *is a 7x7 synaptic weights matrix of a given hidden layer neuron H;* $⨀$ *gives Hadamard (or element-wise) product of two matrixes,* $grand\left( A \right)$ *is a grand sum of all the elements of a matrix* $A$ *(g*$rand\left( A \right)=e^{T}Ae$*, where e is all-ones vector).* $DirectionMask(O)$ *takes in an output layer neuron,* $O$*, and returns a matrix that represents the direction of motion with respect to the input field. For example, when the neuron that directs the critter to move up and to the left is supplied as input, the function returns a 7 x 7 matrix of zeros with the top left 3 x 3 submatrix being ones.* $W_{H\to O}$ *simply returns the synapse strength from the source (*$H$*) to destination (*$O$*) neuron.*

*Although this is seemingly an intricate metric, it captures many desired features of the network’s connectivity and responses to food particles present in the visual field. Conceptually, this metric is similar to the method used for developing the receptive fields of output layer neurons with respect to the input field (Figure 2, 5). PRM builds upon this qualitative visualization, allowing us to numerically assess how specific particles influence output layer neurons to spike when present in the portion of the visual field that corresponds to the direction of motion for that neuron. The intuitions of the metric are as follows:* $W_{H} ⨀ P$ *develops a notion of how well the current hidden neuron’s (*$H$*) connections to the input layer overlaps with the current food particle (*$P$*) placed at specific location. The resulting matrix is then multiplied by* $grand\left( W_{H} ⨀ P \right)^{2}$*, which emphasizes contribution of the H neurons receiving input from adjacent pixels in correct orientation (i.e., sensitive to the food particles) vs those receiving input from random pixels. Indeed, when a hidden layer neuron H overlaps strongly with a food particle P, the chances of spiking are significantly increased, thus this nonlinear term captures the high impact overlapping receptive fields and food particles has on output layer activity.* $W_{H\to O}$ *captures how strongly the current output layer neuron* $O$ *is listening to the current hidden layer neuron* $H$*.*

*These described pieces are multiplied together to form a weighted input receptive field of the output layer neuron with respect to a specific hidden layer neuron and food particle type / location. The sum of these terms for all hidden layer neurons and food particle locations is taken for a single output layer neuron, achieving a global view of all hidden layer neurons and food particle types / locations influencing the current output layer neuron. The* $grand\left( A \right)$ *operation between the* $DirectionMask(O)$ *and the previously described summed term is then taken to see how much the summed weighted receptive fields overlap with the corresponding direction of movement for output neuron* $O$*. This process is repeated for all output layer neurons to get a global quantification of how the current food particle influences activity in the direction of motion for all output layer neurons. When this metric is calculated for a given network state across food particle types we can observe what food particles impact output layer activity and drive the critter to move, highlighting what particle orientations the network is attracted to.”*

Line 173

*“To further quantify the network’s sensitivity to various particle types we developed a metric termed the Particle Responsiveness Metric (PRM) to gauge how specific particles influence activity of the output layer neurons, see the section* ***Methods: Particle responsiveness metric*** *for further details. Using PRM on all food particle orientations across ten trials, we found that following Task 1 training the network is drawn to horizontal particles (Figure S5 A) while post Task 2 training vertical particles drive output layer activity (Figure S5 B), thus quantitatively supporting the qualitative results displayed in figure 2.”*

Line 298

*“To validate these qualitative results we inspected the PRM metrics for all food particle orientations across ten trials following Interleaved_S,T1_ training. The comparatively high mean values for horizontal and vertical food particle orientations revealed the network’s movement was significantly driven by these rewarded food particle orientations (horizontal and vertical), quantifying multitask memory integration into the network’s synaptic weight matrix. (Figure S5 C).”*

*Line 1086*

*“S5 Figure. Particle responsiveness metric (PRM) shows correspondence between type of training and particles preferred by the network. (A-D) Mean and standard deviation (blue bars and black lines respectively) of the PRM for various types of training and particle orientations across ten trials. The title of each plot reflects the most recently trained stage, the vertical axis corresponds to the value of the PRM while the horizontal axis identifies the particle type (bold labels indicate ideal particles the network would be attracted to following the corresponding training). It can be seen that the metric indicates the network is most responsive to the corresponding ideal particle types following a specific training regime e.g. Post Task 1 the network is most responsive to horizontal particles (A), Post Task 2 the network is most responsive to the vertical particle (B), Post I_S,T1_ the network is most responsive to the horizontal and vertical particles (C), Post I_T1,T2_ the network is most responsive to the horizontal and vertical particles (D).”*

**Figure S5**

With regards to the reviewer minor comments, here are our responses:

1. Fig 1A says “apply to all middle-to-output connections”; what does middle mean here? If middle means hidden layer, better to say “hidden” for consistency and clarity. It is also not clear to me whether the blue and grey are supposed to represent different things, e.g. the blue lines next to “apply to all middle-to-output connections” suggests it is a legend indicating where the global reward signal is applied to; but a lot of the parts in blue do not receive a reward signal.

We have modified the figure to be more clear. We changed “Middle Layer” to be “Hidden Layer”. Gray arrows represent within-network properties such as weights, cyan arrows represent critter to environment action selections, while the orange arrows represent environmental consequences of the action (i.e. value of the reward signal).

**Figure 1 A**

2. Lines 124-125: if I understand correctly from this, performance of 0.7 means 70% of the pairs of particles that the model encountered were “rewarded” and 30% were “punished”. I am not sure this is explained – would be helpful if this was made explicit.

Thank you for mentioning this oversight, we have emphasized the meaning of the performance metric

Line 123

*“In either task, the network learned to discriminate between rewarded and punished particle patterns in order to acquire as much reward as possible. In these simulations we consider pattern discriminability (ratio of rewarded vs punished particles consumed) as a measure of performance, with chance performance being 0.5.”*

3. Lines 139-140 state a standard deviation of 0.2, which is both surprisingly high and inconsistent with what is shown in figure 1; I believe this is a typo which should say 0.02

Thank you, we have corrected the errors.

Line 140

*“After training Task 1, mean performance across ten trials on Task 1 was 0.70 ± 0.02 while performance on the untrained Task 2 was 0.53 ± 0.02 (chance level).”*

4. Fig 1 and text on lines 139,-140, 173-174, 179-180, & 199-201 mention mean and standard deviation of performance; presumably this is over a number of experiments with different random seeds, but this is not made explicit. Please indicate the number of experiments (or otherwise, what is being averaged over).

Yes, this is correct. We have modified the text to specify the number of trials

(see Lines 122, 140, 175, 183, 213, 299, 254, 1057)

5. In fig 3E there is some interesting variability in the performance of different runs. At best (mean + standard deviation), task 1 retesting shows no forgetting at all, while at worst (mean - standard deviation) there is some forgetting with a decrement in performance of about a quarter. It would be useful for the authors to address why this might be. Perhaps in some runs the synaptic weight configurations were more orthogonal, so harder to find an intersection during interleaved training?

This variability did indeed initially strike us as potentially important for additional synaptic weight analysis, but upon digging into the single trial performance results, it appears that the variability is more reflective of the network's initial ability to learn Task 1, rather than reflective of it’s ability to accommodate Task 2. The figure below shows the performance over simulated time for 10 random seeds which compose Fig 3E. As can be seen, the rank ordering of the trials is largely conserved across all testing periods. Focusing on Task 1 testing periods (i.e. time blocks between the 2nd & 3rd and 4th & 5th dashed lines), it appears that most of the trials all performed similarly, with a modest amount of forgetting on Task 1. However, trials 6 (brown) and 10 (cyan) actually improved their performance on Task 1 following sleep. These two trials were also those which performed the best on Task 1 initially.

6. In relation to lines 431-436, finding convergent evidence in the literature for the impact of sleep on perceptual learning: these studies (reference 46, in particular) show that sleep is necessary for the initial learning of a task, which appears to be quite different from the conclusion of this manuscript that acquisition of one task does not require sleep, but sequential acquisition of two tasks does. It would be helpful if the authors could be clearer about how they believe their model relates to such findings in this respect.

Thank you for bringing this point of confusion to our attention. We tried to approach this work from the perspective of how complex actual biological learning is (i.e. there are always interfering tasks, either due to previous learning or task-irrelevant, yet novel experiences during and after learning). We model a very controlled version of this, where the first task is learned without any pre-existing memories (i.e. no proactive interference is possible) and there are no post-learning factors that could potentially damage a recently learned task (i.e., no retroactive interference, unless we explicitly train a 2d task subsequently). In this case, the network starts from a blank slate, which is not at all an appropriate comparison to a biological neural network, particularly not for adults, and trained weights remain unchanged post-training unless explicit learning of a new subsequent task affects them. For this reason, we consider the appropriate baseline state of our model to be just after Task 1 training, once there exist some prior memories to serve as a bias to the network. Viewed from this perspective, we believe the results of our study are relevant to the citing literature.

More specifically, the rightmost panels of Figure 3 spell this out the cleanest. Panel B may be compared to a situation when a new task (Task 1 or Task 2) is trained in an experimental setting to great accuracy and tested immediately following acquisition (i.e. no other learning, task-related or otherwise, intervenes between training and testing). Panel D can be compared to a situation when a new task (Task 2) is trained gradually (i.e. with time between experimental sessions when the subject may be re-exposed to previous learned information, Task 1), and without any intervening periods of sleep (i.e. the sleep-deprived condition of the cited works). In this case, we see a reduced Task 2 performance relative to the more controlled setting in Panel B. To discuss this important point, we included the following new text:

Line 475

*“In comparing our modeling results to those found in the literature on biological learning, it is important to note an important difference in the “baseline” state of an animal undergoing an experimental training condition versus a neural network model. In our model, and indeed in all neural network models, the system begins as a “blank slate” without knowledge of any previous learning or competing demands. In contrast, animals under experimental training paradigms have a wealth of experiences which would serve as priors to bias the subsequent learning during training, leading potentially to proactive interference. Moreover, training is typically conducted across multiple days, with intervening periods during which the animal will be subject to an array of various task-irrelevant stimuli and organismal demands possibly leading to retroactive interference. Both of these ensure that the baseline state of the animal entering a given training session is far from that of the “blank slate” a neural network model enters with as well as that recently learned memories may start degrading quickly in the brain while the network weights remain unchanged post training (unless new task is explicitly trained). Due to this stark difference, we focus our attention on the interference phenomena which follow training on an initial task as opposed to initial learning. Viewed from this perspective, initial task training in our network can serve a similar role to the prior personal history of an animal subject.”*

Review #2

Thank you for your feedback on our submission, we have taken the appropriate steps to address your concerns.

“One minor issue that I had was about the interpretation of the results in terms of synaptic weight changes. In principle, it could help intuitive understanding that analysis and explanations are provided both at the level of (the statistics of) single weights and in high-dimensional weight space. However, while I like the explanation of the results in terms of solution manifolds, I find the explanations in terms of single weights a bit simplistic and possibly misleading. In particular, evolution of the weight vector over one of the solution manifolds towards the intersection of the two manifolds does not necessarily entail the preservation of individual strong weights. In fact, if the preservation of strong weights is a key feature of interference-free learning in this network, then it is not clear why the simulation experiment where strong weights were explicitly frozen did not achieve the same goal - although this might depend in further details of training that are not highlighted in the paper.”

We agree with this sentiment that “sleep” is doing more than merely preserving task relevant weights, the maintenance of task relevant synapses is only part of the effects we observe . As you mention above, this sleep-like process performs a complex transformation on the weight matrix that yields much better performance than simply amplifying weights. Indeed, if only one tasks is trained, the network can allocate all available synaptic resources to that task. When few tasks compete for the same resources, simply freezing the strongest weights of the earlier task may not be ideal solution since some strong weights may be redundant to support that task and some may be needed for another tasks. So, ideal solution may require some “intelligent” synaptic weights adjustments that is hard to do manually but may be happening naturally during spontaneous unsupervised replay. As such, we have modified the manuscript to emphasize this point.

Line 343

*“Thus, in all cases, some degree of retroactive or prospective interference was observed highlighting the fact that the sleep-like stage performs a significantly more sophisticated modification to the weight matrix than simply freezing (or amplifying) task relevant synapses. Sleep is capable of intelligently selecting certain strong synapses to maintain in addition to which weak synapses should be strengthened. Indeed, the sleep phase results in a large cluster of weights being renormalized around an intermediate value of synaptic strength in the network. This may also explain why we observed somewhat better overall performance (combined performance on both tasks) after sleep compare with interleaved training (see Fig. 3). Indeed, interleaved training requires repetitive activation of the entire memory pattern, so if different memory patterns compete for synaptic resources each phase of interleaved training will enhance one memory trace but damage the others. This is in contrast to spontaneous replay during sleep when only task specific subsets of neurons and synapses may be involved in each replay episode.”*

“Although the high-level results achieved by the agent are quite convincing, I would have been interested in learning more about what is going on "under the hood". In particular, none of the figures shows any examples of activity in the network either at the single neuron or at the population level (e.g., voltage traces or spike rasters). I think it would be useful to see illustrations of network activity (and perhaps related synaptic changes) in both the awake and the sleep phase of training.”

We have added plots of activity in the supplemental figures to give insight on how the model behaves in terms of spiking (Figure S6).

**Figure S 6**

Line 1109

“**S6 Figure. Spike rasters showing network activity across various training regimes. (A-D)** Representative spike rasters from various training regimes. The vertical axis specifies a unique neuron in the network while time in epochs is shown horizontally. Here a single dot represents a specific neuron spiking at a given time while the color of the dot dictates what layer that neuron belongs to (green, blue, red corresponding to input, hidden, and output layers respectively). Panels A, B, C, D correspond to sample activity from Task 1 training, Task 2 training, I_T1,T2_ training and I_S,T1_ training respectively. Note, during I_S,T1_ there are alternating periods of Task 1 training and sleep, in panel D activity is taken during a period of sleep when the hidden layer is spontaneously activated. Thus, there are hidden (blue) and output (red) layer spikes while the input (green) layer is completely silent.”

With respect to your minor critiques, are response are below:

- The figures show time in units of "aeons". Beside the fact the this word normally indicates extremely long periods of time (in some contexts, a billion years), this unit is not defined in the Methods section, and it is not clear what it corresponds to (either in simulation time steps or in real time).

We have added the definition of aeons to the methods section.

Line 559

*“Each simulation consisted of millions of these movement cycles / epochs, therefore a unit of time was introduced termed aeon (1 aeon = 100 epochs) for concise reporting.”*

- I think that the graphical representation of the solution manifolds in Figure 7 is a bit misleading. In particular, as the solution manifolds of the individual tasks are depicted as 2D surfaces, their intersection should be a 1D (closed) curve not a 2D region.

Thank you for highlighting this point of confusion, we have corrected the text to reflect that the manifolds are three dimensional volumes whose boundaries are defined by the surfaces depicted.

Line 393

*“In Figure 7G we present a schematic depiction of these results. The task-specific manifolds, M_T1_ and M_T2_, are depicted in 3D as two volumes whose boundaries are defined by two orthogonal elliptic paraboloids with opposite orientation. The ellipsoidal intersection approximates the volume comprising M_T1∩T2_.”*

Line 866

*“(G) Cartoon illustration of the task-specific point-sets shown in (A-C) as solution manifolds M_T1_ (red) and M_T2_ (blue). M_T1_ and M_T2_ can be thought of as two volumes with boundaries defined by the interiors of oppositely oriented elliptic paraboloids which intersect orthogonally defining an approximately ellipsoidal volume near the origin (M_T1∩T2_; dark green).”*

- Have you tried to estimate the dimensionality of the solution manifolds? If so, does this provide a hint about the capacity of the network for learning multiple tasks?

This is very interesting question and something we would like to explore more but we are not sure what would be a best way of doing it, and it may led to extensive analysis that would go beyond the scope of our initial work. Other works have suggested that the latent dimensionality of solution manifolds is mainly determined by task complexity (Gao et al, 2017, bioRxiv; Jazayeri & Ostojoc, 2021, arXiv) as opposed to encoding dimensionality. Indeed, our task has essentially 3 dimensions to it (i.e. possible movement directions). Using a simple measure of dimensionality, the participation ratio

$$PR=\frac{\left( \sum_{i} \lambda_{i} \right)^{2}}{\sum_{i} \lambda_{i}^{2}}$$

where each 𝜆*_i_* is a normalized eigenvalue of the covariance matrix, and the eigenvalues taken from performing a PCA on our synaptic weight data (see figure below for top 3 eigenvalues) we can compute a dimensionality of ~3.4 for our synaptic weight space. However, this approach may be oversimplified and subject to various critiques, so we may prefer avoid reporting it in the manuscript.

- In line 548, there is a term (1-R+2XR) which does not appear in the equations, and may correspond to an alternative formulation of noise in which X is sampled from [0,1] rather than [-1,1].

Thank you for your comment, the equation has been updated to reflect the correct value of (1-R+2XR) and the sample range of X and been corrected to [0,1]

Line 595

Line 601

*“The standard value of R is 0.12. X is a random variable sampled from a uniform distribution with range [0, 1].”*

- In lines 650 and 653, M should probably be M_\tau

This is correct, thank you for pointing this out. We have corrected the text.

Line 709

*“Here, d n is the n-dimensional Euclidean-distance function, where n is the dimensionality of synaptic weight space (i.e. n = 6272 here),* $M_{\tau}$ *is the point-set specific to the manifold or intersection in question (i.e. either M_T1_, M_T2_, or M_T1∩T2_), and x is a particular element of the point-set* $M_{\tau}$*.”*

- The description of synaptic interactions in the Methods is a bit unclear. In particular, how is g_syn related to the W's? And what is the temporal evolution of WI_i,j? Is it subject to plasticity?

We are sorry that we did not write this clearly. The variable **g_syn** (Line 595) in the model is directly proportional to the synaptic strength W (so that weak synapse delivers small g_syn and vice versa). We did not focus on the role of inhibition in this model, so the inhibitory connections are very simple. They are not subject to plasticity and their temporal evolution just follows their excitatory opponents to counterbalance them in average. We have added the equation that dictates their values through time.

Line 654

*“Additionally, all inhibitory synapses were modified via a similar heterosynaptic update rule following each STDP event where the strength of every outgoing inhibitory weight from a given neuron was set to the negative mean of all outgoing excitatory synapses of that same neuron. More rigorously:”*

$${WI}_{ij}\underset{\leftarrow}{}{-\frac{1}{|j|}\sum_{j} W_{ij}}$$

- There are several typos and grammatical errors that should be corrected.

Thank you. We have thoroughly reviewed the text and believe we have corrected all of these errors.
