# Supplementary material for: Sleep prevents catastrophic forgetting in spiking neural networks by forming a joint synaptic weight representation
Source: PLoS Comput Biol. 2022 Nov 18;18(11):e1010628. doi: 10.1371/journal.pcbi.1010628 (PMC9674146; doi:10.1371/journal.pcbi.1010628)
Supplement: Attachment — Submitted filename: PlosCompBioCommentsOfficalResponse-9-26-22.docx [file pcbi.1010628.s008.docx]

Reviewer #1,

Thank you for your comments on our manuscript, in response to your comments, please see the following:

“The text regarding the particle responsiveness metric refers to figure S5, when the relevant figure is S3.”

The document references figure S5 once on Line 284. Here the text is referring to Interleaved_T1,T2_ and Figure S5 displays the corresponding analysis, it is not meant to refer to the PRM. If there was perhaps another location where a figure was incorrectly referenced, please let us know.

The text where we discuss PRM is correctly referring to fig S3, as reviewer expected, please see text below:

*“To further quantify the network’s sensitivity to various particle types we developed a metric termed the Particle Responsiveness Metric (PRM) to gauge how specific particles influence activity of the output layer neurons (see the section Methods: Particle responsiveness metric for further details). Using PRM on all food particle orientations across ten trials, we found that following Task 1 training the network is drawn to horizontal particles (Figure S3 A) while post Task 2 training vertical particles drive output layer activity (Figure S3 B), thus quantitatively supporting the qualitative results displayed in Figure 2. “*

And

*“To validate these qualitative results we inspected the PRM metrics for all food particle orientations across ten trials following InterleavedS,T1 training. The comparatively high mean values for horizontal and vertical food particle orientations revealed the network’s movement was significantly driven by these rewarded food particle orientations (horizontal and vertical), quantifying multitask memory integration into the network’s synaptic weight matrix. (Figure S3 C). “*

“The sentence on line 63 of supplementary figure legends appears to end prematurely.”

Thank you, we have appended the missing text.

Reviewer #2,

Thank you for your feedback, it has helped to greatly improve our manuscript.
